# The Interplay Between Monocytes/Macrophages and CD4^+^ T Cell Subsets in Rheumatoid Arthritis

**DOI:** 10.3389/fimmu.2015.00571

**Published:** 2015-11-19

**Authors:** Ceri A. Roberts, Abigail K. Dickinson, Leonie S. Taams

**Affiliations:** ^1^Centre for Molecular and Cellular Biology of Inflammation (CMCBI), Division of Immunology, Infection and Inflammatory Disease, King’s College London, London, UK

**Keywords:** rheumatoid arthritis, inflammation, immune regulation, cell polarization, myeloid cell, T helper cell, Treg

## Abstract

Rheumatoid arthritis (RA) is a chronic inflammatory disease characterized by inflammation of the synovial lining (synovitis). The inflammation in the RA joint is associated with and driven by immune cell infiltration, synovial hyperproliferation, and excessive production of proinflammatory mediators, such as tumor necrosis factor α (TNFα), interferon γ (IFNγ), interleukin (IL)-1β, IL-6, and IL-17, eventually resulting in damage to the cartilage and underlying bone. The RA joint harbors a wide range of immune cell types, including monocytes, macrophages, and CD4^+^ T cells (both proinflammatory and regulatory). The interplay between CD14^+^ myeloid cells and CD4^+^ T cells can significantly influence CD4^+^ T cell function, and conversely, effector vs. regulatory CD4^+^ T cell subsets can exert profound effects on monocyte/macrophage function. In this review, we will discuss how the interplay between CD4^+^ T cells and monocytes/macrophages may contribute to the immunopathology of RA.

## Rheumatoid Arthritis

Rheumatoid arthritis (RA) is a chronic inflammatory and debilitating disease, characterized by inflammation of the lining of the joint (synovitis), eventually leading to the destruction of cartilage and the underlying bone. RA affects between 0.5 and 1% of the Western adult population with a female:male ratio of 3:1. The exact etiology of RA is still unknown, but it is widely accepted that RA is a multifactorial disease with genetic, environmental (e.g., smoking), gender and age-associated factors contributing to the disease process (1–3).

Typical hallmarks of RA are pannus formation and synovial hyperplasia, caused by proliferating fibroblasts and infiltrating immune cells. These events promote leukocyte recruitment, immune cell activation, and production of inflammatory mediators and proteinases, all of which eventually contribute to joint damage. A wide range of immune cells has been detected in the RA joint, including CD4^+^ T cells, CD8^+^ T cells, B cells, NK cells, γδ T cells, mast cells and myeloid cells. Various soluble mediators produced by these immune cells have been shown to correlate with disease progression and/or severity in RA, e.g., rheumatoid factor, anti-citrullinated peptide antibodies, tumor necrosis factor (TNF)α, interleukin (IL)-6, IL-1, and IL-17A (2, 4). The importance of the immune system in disease pathogenesis is illustrated by the recent success of biologic therapies that target key inflammatory cytokines (e.g., TNFα blockade and anti-IL-6R therapy), immune molecules (e.g., CTLA4-Ig leading to blockade of CD80/CD86-mediated costimulation), and immune cells (e.g., B cell depletion).

## Monocytes and Macrophages in Rheumatoid Arthritis

Monocytes/macrophages are a potent source of proinflammatory cytokines, in particular TNFα, IL-6 and IL-1, and matrix metalloproteinases (MMPs), leading to endothelial cell activation, acute phase reactions, and cartilage damage. These cells can also produce a wide range of chemokines, which help recruit additional leukocytes to the inflamed joint. In addition, monocytes have the ability to polarize CD4^+^ T cells and can differentiate into osteoclasts, which may further contribute to their role in RA pathogenesis. As such, monocytes and macrophages are viewed as relevant therapeutic targets in RA (5–7).

Myeloid cells with a monocyte/macrophage phenotype (i.e., CD14^+^CD68^+^) are present in large numbers in the rheumatoid joint. Several studies have shown that these cells produce proinflammatory cytokines (8, 9) and have an activated phenotype with increased expression of HLA-DR (involved in antigen presentation to CD4^+^ T cells), costimulatory molecules (e.g., CD80, CD86, and CD40), adhesion molecules (e.g., CD54), and some chemokine receptors (10–18). The importance of synovial CD68^+^ macrophages in RA pathogenesis is underlined by the findings that the presence of these cells correlates with disease activity markers (19, 20) and that a change in their presence has been reported to be a reliable biomarker for response to treatment (21). An early, small study in patients with RA (*n* = 10) noted that synovial CD68^+^ cells were reduced in the perivascular and connective tissue areas 12 weeks after treatment with gold (22). Sublining macrophage count was also shown to correlate significantly with radiologic outcome and radiologic progression in patients with RA (*n* = 23–27) (20). An elegant study by Haringman et al. investigating arthroscopic synovial tissue biopsies from patients with RA (*n* = 88) participating in various clinical trials, showed that a reduction in the number of synovial sublining CD68^+^ macrophages correlated significantly with clinical improvement independently of the therapeutic strategy (23). Importantly, the number of sublining macrophages did not change after placebo or ineffective treatment, supporting its use as a predictive biomarker for response to treatment (21, 23).

A growing number of studies have reported on the frequencies and phenotype of peripheral blood monocyte subsets in RA. Phenotypically, peripheral CD14^+^ monocytes in patients with RA show some signs of altered activation with studies reporting increased expression of CD14, FcγRs, CD54, CD11b, and/or HLA-DR (18, 24–27), although not all studies agree on increased HLA-DR expression (24, 27). Peripheral blood monocytes from RA patients are also reported to express increased levels of transmembrane TNF (tmTNF) (28). Human monocytes can be divided into subpopulations based on expression of CD14 [lipopolysaccharide (LPS) coreceptor] and CD16 (FcγRIII). CD14^++^CD16^−^ “classical” monocytes are the most prevalent subset, representing ~90% of blood monocytes in healthy individuals. CD16-expressing cells are less frequent among circulating monocytes but are expanded in infection and inflammatory conditions (29). CD16^+^ monocytes can be further subdivided into CD14^++/bright^ (also called “intermediate”) and CD14^dim^ (“non-classical” or “patrolling”) subsets (29, 30). Earlier studies, which did not necessarily discriminate between CD14^++/bright^CD16^+^ and CD14^dim^CD16^+^ cells, showed an increase in the percentage of CD14^+^CD16^+^ monocytes in RA blood (31–33). CD14^+^CD16^+^ monocytes in RA express high levels of CCR1, CCR5, ICAM-1, or TLR2 (27, 32, 33). Higher frequencies of CD14^+^CD16^+^ blood monocytes were associated with clinical parameters of active disease (31, 33). More recent data suggest that only the frequency of CD14^++/bright^CD16^+^ intermediate cells is increased in peripheral blood of patients with chronic RA compared to sex- and age-matched healthy donors, while the frequency of non-classical CD14^dim^CD16^+^ monocytes does not differ between patients and controls (27, 34, 35). The frequency of CD14^++/bright^CD16^+^ cells at baseline was found to negatively correlate with response to methotrexate treatment (34). However, another study reported, using absolute cell counts, that both CD14^++/bright^CD16^+^ and CD14^dim^CD16^+^ monocyte populations are increased in RA blood (36). Ligation of CD16 using immune complexes or FcγRIII-specific mAb enhances proinflammatory TNFα production, suggesting that the expression of CD16 could be functional (32, 34, 37).

Recently, Krasselt et al. showed that CD14^bright^CD56^+^ monocytes, which are predominantly composed of classical CD14^bright^CD16^−^ cells, produce more TNFα, IL-10, and IL-23 in response to LPS and demonstrate increased spontaneous production of reactive oxygen intermediates [reactive oxygen species (ROS)] compared to CD14^bright^CD56^−^ monocytes (38). CD14^bright^CD56^+^ frequencies were positively correlated with age in healthy controls and were expanded in the blood of young RA patients compared to age-matched healthy controls. The CD14^bright^CD56^+^ subpopulation was reduced in a longitudinal study of 16 RA patients following anti-TNF treatment. Additional studies are required to further elucidate the role of CD14^bright^CD56^+^ monocytes in RA.

In RA synovial fluid, it appears to be the intermediate CD14^++/bright^CD16^+^ monocyte population that is increased in frequency compared to matched peripheral blood (31, 33, 35). One explanation for the increased frequency of CD16 expressing synovial monocytes is that there is a specific expansion of the CD14^++/bright^CD16^+^ monocyte population. Another non-mutually exclusive explanation is that there is *de novo* expression of CD16 triggered by the inflammatory milieu. It was shown that *in vitro* stimulation of healthy monocytes with recombinant transforming growth factor β (TGFβ) or RA synovial fluid induced elevated CD16 expression, an effect that was inhibited by TGFβ signaling blockade (35).

Table 1 summarizes the reported phenotypic features of CD14^+^ cells derived from RA peripheral blood or synovial fluid, and cells with a macrophage phenotype in synovial tissue. It should be noted that studies on synovial fluid or synovial tissue generally involve the whole CD14^+^ and/or CD68^+^ population (which may contain monocytes and macrophages), rather than sorted subsets. Therefore, Table 1 represents a summary of relevant literature reports on monocyte/macrophage cell phenotypes *within* different anatomical compartments rather than a direct comparison of these cells *between* different compartments.

**Table 1:** **Phenotypic features of monocytes/macrophages from RA peripheral blood, synovial fluid, and synovial tissue**.

Compartment	Phenotype	Reference
(a) Peripheral blood	↑ HLA-DR, CD14, CD40, CD54, CD11b ↑ Fcγ receptors (including CD16)	(18, 24–26) (18, 24, 26, 27, 31–36)
	↑ CCR3, CCR4, CCR5	(15)
	↑ tmTNFα	(28)
	↑ Spontaneous IL-1β production	(25, 28)
	↑ Resistance to apoptosis	(28, 39)
(b) Synovial fluid	↑ HLA-DR, CD40, CD54, CD80, CD86, CD276	(11, 13, 14, 16–18, 35)
	↑ CD16^+^	(18, 31, 33, 35)
	↓ CCR1, CCR2, CCR4, ↑CCR3, CCR5	(15)
	↑ Resistance to apoptosis	(39, 40)
(c) Synovial tissue	HLA-DR^+^ CD68^+^	(10, 14) (15, 19–23)
	CD163^+^	(12)
	IL-1^+^, TNFα^+^, IL-6^+^, GM-CSF^+^, TGFβ^+^	(8, 9, 14)

*Arrows indicate phenotypic alterations observed in CD14^+^ cells from the peripheral blood of rheumatoid arthritis (RA) patients in comparison to healthy donor peripheral blood CD14^+^ cells (a), or in CD14^+^ cells from RA synovial fluid compared to RA peripheral blood CD14^+^ cells (b). Phenotypic markers that are expressed on cells with a macrophage phenotype from RA synovial tissue (c)*.

## Effect of Monocytes/Macrophages on CD4^+^ T Cell Subsets

In addition to the innate effector functions of monocytes/macrophages in terms of proinflammatory cytokine and chemokine production, their inflammatory role in RA pathogenesis may stem from their function as a bridge to the adaptive immune system. Colocalization of CD14^+^ cells with clusters of CD4^+^ effector T cells at sites of inflammation has been reported in inflamed rheumatoid synovium, as well as in inflamed tonsil, and psoriatic or atopic dermatitis skin (41, 42), suggesting that CD4^+^ T cells and monocytes/macrophages can interact *in vivo* at sites of inflammation.

### CD4^+^ T Helper Cell Polarization by Monocytes/Macrophages

Dendritic cells (DCs) are classically considered to be the major drivers of CD4^+^ T helper cell polarization; however, evidence is accumulating that monocytes/macrophages can also play a role in this process. Monocytes and/or macrophages can be major sources of IL-1β, IL-6, IL-12, and IL-23, cytokines known to be present in the RA joint (4, 8, 9, 43, 44). IL-12 is involved in driving CD4^+^ T helper 1 (Th1) cell polarization, while IL-1β, IL-6, and IL-23 can drive and maintain Th17 polarization. Interferon γ (IFNγ)^+^CD4^+^ T cells (indicative of Th1 cells) and IL-17^+^ CD4^+^ T cells (indicative of Th17 cells) are readily detectable in the RA joint, in both the tissue and the fluid (45–47). Th1 cells were originally thought to be one of the major contributors in RA pathogenesis, based on their abundance in RA synovial fluid, their key role in certain experimental models of arthritis, as well as the inflammatory function of IFNγ particularly on macrophage activation. However, studies have shown that IFNγ may also have a protective, rather than an exacerbating role in RA (48–50), which may be due to its antagonistic effects on Th17 induction (51) or on VEGF production (46, 52), thereby possibly inhibiting angiogenesis.

In recent years, IL-17 and Th17 cells have gained attention as critical mediators in RA pathogenesis. IL-17 is a potent proinflammatory cytokine that works in synergy with TNFα to induce the inflammatory events and joint damage that are characteristic of RA (53, 54). The receptors for IL-17 (IL-17RA and IL-17RC) are expressed in RA synovium, including on CD14^+^ monocytes/macrophages (55) and stimulation of RA synovium with IL-17 leads to production of IL-6, MMPs, and joint degradation (56–58). Blood CD14^+^ monocytes can be potent inducers of human Th17 responses depending on their activation status. Human blood monocytes activated by peptidoglycan or LPS were shown to efficiently promote Th17 responses from cocultured naive CD4^+^ T cells in the presence of anti-CD3 mAb (59). Our own lab found that following *in vitro* activation with LPS, peripheral blood CD14^+^ monocytes from either healthy donors or RA patients promoted Th17 responses in an IL-1β- and TNFα-dependent manner (17, 60). It was also shown that human monocytes stimulated *in vitro* with heat-killed pneumococci triggered a Th17 response which was dependent on TLR2 signaling (61). In contrast, stimulation with live pneumococci led to a mixed Th1/Th17 response due to monocyte production of IL-12p40. In a non-infectious setting, peripheral blood monocytes from patients with type 1 diabetes spontaneously secreted the proinflammatory cytokines IL-1β and IL-6. These cells induced higher frequencies of Th17 cells from memory T cells *in vitro* compared with monocytes from healthy control subjects, which was reduced by a combination of an IL-6-blocking Ab and IL-1R antagonist (62). Finally, healthy peripheral blood monocytes that were treated with RA synovial fluid prior to coculture with anti-CD3/CD28-stimulated CD4^+^ T cells were shown to promote Th17 differentiation, which was attributed to a TNFα-mediated increase in monocytic production of IL-6 and IL-1β (63).

Additional studies in mice and human show that monocytes/macrophages from the synovial fluid of the inflamed arthritic joint, which may contain extravasated monocytes as well as tissue-resident macrophages, can promote IL-17 production in CD4^+^ T cells (17, 35, 64). These data suggest that newly recruited CD4^+^ T cells in the rheumatoid joint might be steered toward a Th17 response by local monocytes/macrophages. The ensuing positive feedback loop between Th17 cells and monocytes/macrophages may then perpetuate inflammation (42). In our own work, the induction of Th17 responses by *in vivo* activated monocytes isolated from RA synovial fluid was found to be independent of TNFα, IL-1β, IL-6, and IL-23, and to involve cell contact (17). In line with our observations, Yoon et al. found increased Th17 responses when stimulated autologous peripheral blood memory CD4^+^ T cells were cocultured with RA synovial fluid monocytes, compared to responses elicited by circulating monocytes. In contrast to our finding that Th1 polarization was not strongly affected by the anatomical origin of monocytes (17), Yoon et al. found that intracellular IFNγ expression (and in most donors tested, the level of IFNγ in culture supernatants), was also significantly increased by coculture with synovial monocytes compared to blood monocytes (35). Thus, synovial monocytes/macrophages may promote both Th17 and Th1 responses.

It is currently unclear whether the capacity of monocytes to promote Th17 responses resides in a particular monocyte subset. Rossol et al. found that circulating intermediate CD14^++/bright^CD16^+^ monocytes in the presence of LPS promoted Th17 cell expansion from peripheral blood memory CD4^+^ T cells *in vitro*, and that the frequency of CD14^++/bright^CD16^+^ monocytes in peripheral blood of RA patients correlated closely with *ex vivo* Th17 cell frequencies (27). Traunecker et al. reported that non-classical (CD14^dim^CD16^+^) monocytes from healthy donors when cocultured with autologous CD4^+^ T cells and specific superantigens in the absence of pathogen-recognition receptor (PRR) stimuli were more efficient stimulators of IL-17-producing T cells; however, in the presence of PRR stimuli, Th17 expansion was mostly observed in cocultures with classical (CD14^++/bright^CD16^−^) or intermediate (CD14^++/bright^CD16^+^) monocytes (65). Blocking of LFA-1/ICAM-1 interaction increased the frequency of IL-17-producing T cells expanded from non-classical monocyte/CD4^+^ T cell/superantigen cocultures. However, no significant differences in capacity to promote Th17 responses were observed in experiments assessing peripheral blood monocyte subsets from 11 RA patients. In contrast to the above studies, Smeekens et al. found that only CD14^++/bright^CD16^−^ classical monocytes and not CD16^+^ monocytes could induce a protective Th17 response in response to *Candida albicans*, due to increased IL-1β and prostaglandin E2 production (66). It is crucial to achieve high degree purity separation for functional characterization of cell subpopulations and the use of magnetic bead separation to isolate CD16^+^ vs. CD16^−^ monocytes rather than high purity FACS-based cell sorting in the study by Smeekens et al. may therefore limit interpretation of the results. Nonetheless, a recent study by Liu et al. also found that after coculturing autologous memory CD4^+^ T cells with FACS-purified monocyte subsets and anti-CD3, classical CD14^++/bright^CD16^−^ monocytes most potently expanded IL-17^+^ memory CD4^+^ T cells (67). Non-classical CD14^+^CD16^++^ monocytes were the strongest inducers of IFNγ expression in naive CD4^+^ T cell cocultures. The above *in vitro* experiments employ different modes of T cell activation with or without PRR stimulation of monocytes, which may contribute to the heterogeneity of the results obtained. Activation of T cells via crosslinking of soluble anti-CD3 (67) triggers MHC-independent T cell activation, whereas stimulation via superantigen (65) crosslinks MHC on monocytes and TCR on T cells, more closely representing an MHC-restricted antigen-specific stimulation. Altering the activation state of monocyte subsets via PRR agonists (27, 65, 66) may also affect their capacity to polarize CD4^+^ T cells. Any conclusions drawn from these and future studies regarding the contribution of different monocyte subsets to CD4^+^ T cell polarization should take into account the choice of *in vitro* stimulation and ideally should be confirmed in multiple systems.

Monocytes/macrophages are also a major source of IL-15 (68), a pleiotropic cytokine which mediates several important proinflammatory effects on both monocytes/macrophages and CD4^+^ T cells. The IL-15Rα is overexpressed on blood-derived lymphocytes and monocytes in RA patients (69), and IL-15 is found at high levels in RA SF (70). IL-15 plays an important role in regulating T cell migration and was shown *in vivo* to facilitate accumulation of adoptively transferred T cells in RA synovial tissues engrafted into immune deficient SCID mice (71). IL-15 also promotes TNFα production by synovial T cells (72), and IL-15-activated blood-derived or synovial T cells can induce TNFα in a macrophage cell line and in RA blood- or synovium-derived monocytes/macrophages in a cell-contact-dependent manner (72). IL-15 may also promote IL-17 production by synovial T cells (73), although in mice a fine-tuning effect of IL-15 on Th17 differentiation was reported (74). IL-18 is another proinflammatory cytokine expressed in RA synovium, most prominently in CD68^+^ macrophages (75). IL-18 acts in synergy with other cytokines, including IL-12 and IL-15, to stimulate T cell production of IFNγ and synovial macrophage release of TNFα (75). In the RA joint, IL-18 also acts as a chemoattractant for synovial CD4^+^ T cells (76) and monocytes (77). Together, these data indicate that production of IL-15 and IL-18 by monocytes and/or macrophages may also be relevant in driving or polarizing inflammatory CD4^+^ T cell responses in the RA joint.

In addition to their role as antigen-presenting and cytokine-producing cells, macrophages can efficiently generate ROS, an important antimicrobial defense mechanism that occurs via activation of the phagocytic NADPH oxidase complex. Rats and mice with genetic variation in the neutrophil cytosolic factor *Ncf1* (encoding p47phox, an activating protein in this complex) demonstrate reduced capacity to exert oxidative burst and increased incidence and severity of T cell-dependent arthritis (78, 79). When efficient ROS production was restored specifically in macrophages, T cell-dependent arthritis development was ameliorated (80). *In vitro*, T cells from mice lacking functional macrophage *Ncf1* demonstrated increased proliferation and cytokine production in response to antigenic stimulation, as compared to T cells from mice with ROS-competent macrophages. Coculturing T cells from ROS-deficient mice with ROS-competent macrophages led to suppressed T cell IL-2 production and proliferative responses to antigen, suggesting that macrophage ROS production affects antigen presentation *in vitro*. However, irrespective of whether macrophages used in the coculture could produce ROS, IFNγ production was increased when CD4^+^ T cells were derived from ROS-deficient mice, indicating that *in vivo* exposure to macrophage ROS production may suppress Th1 responses. In agreement with these findings, King et al. found that human T cells stimulated with anti-CD3/CD28 in the presence of ROS demonstrated an increase in type 2 cytokines with no alteration in type 1 cytokine production, even in culture conditions polarizing toward a Th1 phenotype, and which could be reversed by concomitant antioxidant exposure (81). Together, these findings suggest that ROS production by macrophages may have the potential to downregulate or modulate T helper cell responses.

It is well known that certain inbred mouse strains show a bias toward either Th1 or Th2 responses, e.g., C57BL/6 and BALB/c, respectively. The bias in Th1- or Th2-like cytokine profiles is maintained in NUDE or SCID mice lacking an adaptive immune system, suggesting a role for the innate immune system in driving this polarization and giving rise to the concept of M1 and M2 macrophages (82). Studies of transgenic (Tg) mice have provided *in vivo* evidence that inflammatory macrophage subpopulations can polarize CD4^+^ T cell responses in mouse models of arthritis. Li et al. crossed a *Floxed* STOP-human/mouse DR5 Tg mouse with the LysM.Cre mouse, to express the Tg human/mouse-chimeric death receptor 5 (DR5) restrictively in myeloid lineage cells. Treatment of these mice with an antihuman DR5 agonistic antibody led to targeted depletion of CD11b^high^Ly6C^+^ inflammatory macrophages and reduced development and severity of collagen-induced arthritis (83). Interestingly, the depletion of this macrophage subpopulation also significantly reduced protein levels of IL-6 and IL-17A in sera, reduced synovial *Il17a*, *Il6*, *Tnfa*, and *Il23a(p19)* mRNA expression and reduced the frequency of IL-17A^+^ and IFNγ^+^CD4^+^ T cells while increasing CD4^+^Foxp3^+^ cells in draining lymph nodes. These data support the notion that macrophages may be important contributors to CD4^+^ T cell polarization.

Dissecting the precise role of monocytes/macrophages in initiating adaptive immune responses can be challenging due to overlapping function and lineage marker expression with DCs. DCs are professional antigen-presenting cells that play a key role in promoting an effective immune response. A multitude of pattern recognition receptors allows DCs to sense invading pathogens and present both exogenous and endogenous antigens to naive and memory T cells. Different subsets of DC exist, including conventional or classical DC (cDC) type 1, cDC type 2, plasmacytoid DC, and monocyte-derived DC (mo-DC). cDC develop independently from monocytes and originate from a common DC progenitor which expresses flt3 (84). It can be difficult to distinguish monocytes or macrophages from mo-DCs or cDC type 2, due to overlap in certain markers (e.g., CD14, CD11b, CD11c, and CX3CR1) (85). CD64 has been identified as a marker that can be used to distinguish mo-DCs from cDCs in mice; however, these cells remain difficult to distinguish from cDCs in humans (86). Recently, researchers have taken a genetic approach to ablate monocytes and macrophages while sparing cDCs and lymphocytes by depleting M-CSF-R^+^LysM^+^ cells. Although neither monocytes nor macrophages were required to initiate immunity, when both cell types were depleted during infection with the intestinal pathogens *Citrobacter rodentium* or *Listeria monocytogenes*, IFNγ^+^CD4^+^ T cells were reduced in the lamina propria, demonstrating the capacity of monocytes/macrophages to influence T cell polarization (87). In contrast, the IL-17 response was not altered by monocyte and macrophage depletion but was significantly impaired upon selective depletion of cDCs, indicating that this cell population is necessary for mucosal Th17 responses. Macrophage depletion has also been shown to reduce type 2-dependent immune responses in the lung and gut. Depletion of CD11b^+^ F4/80^+^ macrophages led to a significant reduction in recruitment and cytokine expression of Th2 cells in affected tissues of three models of IL-13-dependent inflammation, fibrosis, and immunity, without any such reduction in the draining lymph nodes (88). Since CD11b^+^ DCs might also be susceptible to depletion, studies of CD11c-DTR and CD11b/c-DTR double Tg mice confirmed that macrophages but not CD11c^+^ DCs were critical for the maintenance of type 2-dependent responses. Reduced expression of the Th2 cell chemoattractants CCL1 and CCL22 may be one mechanism through which Th2 cell recruitment is impaired in a *Schistosoma mansoni* egg-induced lung granuloma model following macrophage depletion (88). Macrophage production of the chemokine CCL5 was recently shown to be important for the maintenance of stable tissue-resident memory IFNγ-producing CD4^+^ T cell (T_RM_) populations in memory lymphocyte clusters in a mouse model of genital herpes (89). Further studies are needed to investigate the presence of T_RM_ in RA synovial tissue and the requirement for monocytes/macrophages in maintaining these populations.

### T Helper Cell Recruitment by Monocytes/Macrophages

As alluded to the previous section, monocytes/macrophages can play an important role in recruiting or maintaining CD4^+^ T cells in the arthritic joint. The C–X–C motif chemokine receptor CXCR6^+^ is abundantly expressed on type 1 polarized effector memory T cells in RA synovial fluid (90, 91). Expression of CXCR6 on synovial T cells is reported to coincide with elevated expression of CXCL16 (the ligand for CXCR6) by synovial macrophages, endothelial cells, and fibroblast-like synoviocytes (FLS) in hypertrophic RA synovium (90). Significantly increased levels of cleaved CXCL16 have been demonstrated in RA SF compared to control samples (90, 92), and RA synovial tissue macrophages express both CXCL16 and CXCR6 (92). *In vitro* migration experiments demonstrate that healthy PBMC or CXCR6^+^ T cells isolated from RA SF can migrate in response to exogenous CXCL16 or CXCL16 present in SF (90, 92). Together, these data suggest that increased CXCL16 expression in RA synovium, either due to increased expression by macrophages (90) or due to increased influx of monocytes (93), promotes recruitment of CXCR6^+^ T cells and may thereby contribute to synovial inflammation and immunopathology. CXCL16 expression can be differentially regulated by cytokines: the Th2-like cytokines IL-4, IL-10, and IL-13 suppress secretion of CXCL16 by monocytes/macrophages, while the Th1-associated cytokine IFNγ slightly enhances CXCL16 secretion (93). No modulation of CXCL16 levels was observed upon addition of IL-15, IL-18, or TNFα to monocytes/macrophages. In contrast, earlier work found that exposure of monocytes to TNFα upregulated transmembrane expression and secretion of CXCL16 (90) suggesting that a reduction in synovial TNFα levels might impact on recruitment of CXCR6^+^ T cells to the joint. In a small study of three patients responding to anti-TNF treatment, *in situ* immunohistochemistry demonstrated significantly reduced synovial CXCL16 expression compared to the high expression levels observed pretreatment (90). This observation may be due to reduced monocyte numbers in the joint following treatment, since synovial cellularity is known to be reduced soon after anti-TNF infusion (94–96). Conversely, in three non-responder patients, CXCL16 expression remained high. In accordance with reduced CXCL16 expression in the joint following successful anti-TNF therapy, serum levels of CXCL16 were also decreased after anti-TNF treatment in two cohorts of 23 and 44 RA patients, respectively (97, 98). Thus far, these studies suggest that monocyte infiltration and/or type 1 cytokine production in the RA joint may enhance local production of soluble CXCL16, which may exacerbate local inflammation via recruitment of CXCR6^+^ T cells.

Another chemokine receptor interaction of increasing interest in RA is the C–C chemokine ligand CCL20 [also known as macrophage inflammatory protein-3 alpha (MIP-3α)] and its C–C chemokine receptor CCR6 [recently reviewed in Ref. (99)]. CCR6 expression is commonly associated with Th17 cells (100), but this receptor is also expressed on memory T cells (101), including CD4^+^ T cells expressing IFNγ with or without IL-17 (102), as well as DC, B cells, and regulatory T cell (Treg) (99). In the SKG mouse model of T cell-mediated autoimmune arthritis, the CCL20–CCR6 axis is implicated in recruiting Th17 cells to the joint, via spontaneous CCL20 production from adherent FLS (103). The authors found that dispersed monocytes from mouse synovial tissue did not produce CCL20 unless stimulated. Others found that unstimulated FLS from RA patients did not produce CCL20, but that stimulation with either IL-1β or TNFα led to the production of CCL20 at levels sufficient to promote CCR6-specific recruitment of mononuclear cells, and which could be increased by IL-17 and decreased by IL-4 (104). In patients with juvenile idiopathic arthritis, CCL20 mRNA was shown to be constitutively expressed by synovial monocytic cells (105). In addition, *in vitro* exposure to plasmin, a component of the fibrinolytic cascade which is generated in inflamed tissues, can trigger production of CCL20 by human macrophages and lead to chemotactic migration of CCR6^+^ Th17 cells *in vitro* (106). A role for plasmin in RA has been suggested by observations from collagen-induced arthritis (107) but has yet to be confirmed in the human setting. In summary, there is a potential role for monocytes in influencing the CCL20–CCR6 axis in RA.

### Modulation of CD4^+^ Treg Function by Monocytes/Macrophages

In addition to effects on T helper cell polarization, activated monocytes and macrophages can also positively or negatively influence the function of CD4^+^ Tregs through production of soluble mediators. IL-1β has been shown by several groups to drive IL-17 expression by CD4^+^CD25^+^ Tregs [reviewed in Ref. (108)]. Excessive production of IL-6 *in vivo* inhibited inducible Treg generation from naive T cells but did not affect the development and function of natural Tregs (109). A recent study showed that IL-6 negatively affected FOXP3 protein expression by reducing expression of USP7 and disrupting USP7–FOXP3 association. USP7 is a deubiquitinase that prevents proteasomal degradation of FOXP3, thereby increasing FOXP3 expression levels and enhancing Treg function (110). TNFα has also been shown to decrease FOXP3 expression and reduce Treg function (111, 112). Additionally, anti-TNF therapy was shown to induce the differentiation of a suppressive CD4^+^CD25^hi^FOXP3^+^CD62L^−^ Treg subpopulation through conversion of CD4^+^CD25^−^ T cells in RA patients (113).

Contrastingly, TNFα has also been shown to boost Treg expansion and/or function (114, 115). Our lab recently showed that monocytes, activated *in vitro* with LPS or with cytokines known to be present in the RA joint, can induce expression of proinflammatory cytokines (IL-17 and IFNγ) in CD4^+^CD25^+^CD127^low^ Tregs in an IL-6-, TNFα-, and IL-1β-dependent manner (18). However, despite the increase in proinflammatory cytokine expression, these Tregs maintained and even enhanced their suppressive capacity, indicating that acquisition of proinflammatory cytokine expression does not necessarily imply loss of suppressive function (18). Conversely, it has been shown that TNFα and IL-6 can alter the susceptibility of effector T cells to Treg-mediated regulation, making them resistant to suppression (116–118).

Monocytes/macrophages can also boost Treg function directly by producing soluble mediators that are immunoregulatory in nature. A population of CD11b^+^F4/80^+^CD11c^−^ macrophages has been identified in the lamina propria which can induce Foxp3^+^ Treg differentiation through a mechanism dependent on IL-10, retinoic acid, and TGFβ in the local milieu (119). As discussed above, macrophage-derived ROS might play a role in modulating effector T cell responses (80); however, it has also been suggested that ROS can promote Treg-mediated immune regulation (120–122). Using both human and rat systems, macrophages were shown to suppress T cell responses by inducing FOXP3^+^ Tregs in a ROS-dependent manner (120). This was confirmed using macrophages from patients with chronic granulomatous disease (CGD) that are defective in ROS production; CGD macrophages allowed significantly more T cell activation and expansion and induced fewer FOXP3^+^ cells than did macrophages from control subjects. T cells primed by CGD macrophages showed reduced inhibition of responder T cell proliferation and IFNγ or IL-17 production than did cells primed by control macrophages. Similar results were observed using rats with defective ROS production due to a SNP in *Ncf1*.

It was shown that monocyte subsets and their cytokines may have differential effects on subsets of Treg cells. In both humans and mice, ~70% of Tregs express the transcription factor Helios (123). CD16^+^ monocytes have been described to inhibit proliferation of Helios^+^ Tregs through a mechanism dependent on IL-12. In contrast, Tregs lacking Helios expression were suppressed by CD16^−^ monocytes via TNFα, while TNFα blockade specifically expanded the Helios^−^ Treg subset (124).

**Figure 1:**
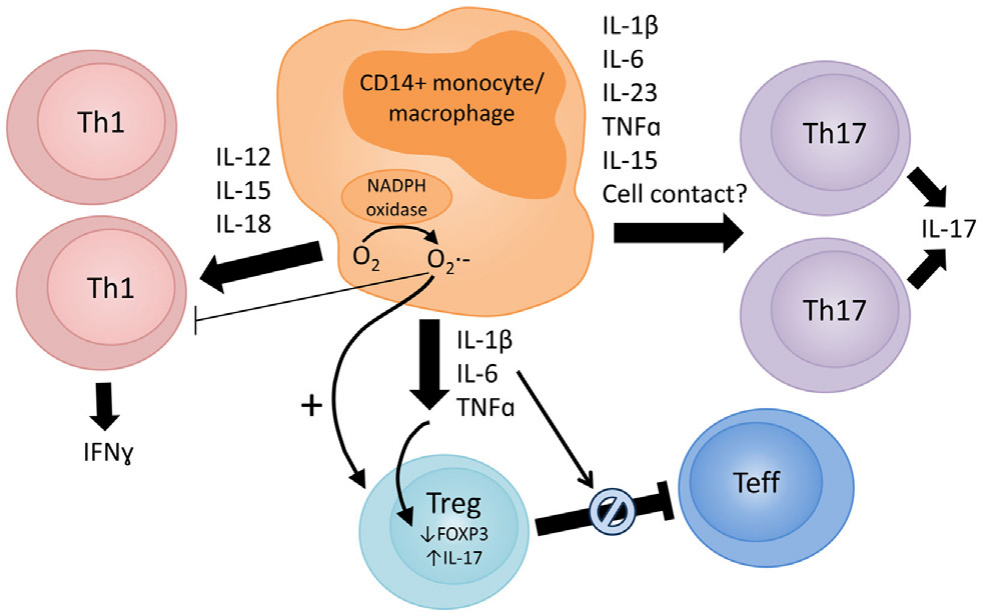
**Proposed mechanisms via which monocytes/macrophages in the inflamed RA joint can modulate CD4^+^ T cell responses**. The arrow thickness reflects the evidence base to support the proposed mechanism. Monocyte/macrophage-derived cytokines, such as IL-1β, IL-6, TNFα, IL-12, IL-15, IL-18, and IL-23, are present in the RA joint (4, 8, 9, 43, 44, 70, 75). These cytokines can promote Th1 (IL-12, IL-15, and IL-18) and Th17 (IL-1β, IL-6, IL-15, IL-23, and TNFα) responses, and both Th1 and Th17 cells are detectable in synovial tissue and fluid (45–47). *In vitro* and *in vivo* data indicate that activated CD14^+^ blood monocytes or synovial monocytes/macrophages can potently induce Th17 (17, 35, 59–64) and/or Th1 (35, 61, 87) responses. Further studies are required to clarify whether different monocyte subsets preferentially promote specific Th responses. Activated monocytes/macrophages can also have profound effects on the phenotype and function of regulatory T cells (Tregs). Several groups have reported that IL-1β can drive IL-17 expression by CD4^+^CD25^+^ Tregs [reviewed in Ref. (108)], but these cells may retain suppressive capacity (18). Others have reported that TNFα and IL-6 lead to reduced FOXP3 expression in Tregs (110–112) or render effector T cells resistant to Treg-mediated suppression (116–118). Macrophage production of reactive oxygen species (ROS) may play a role in suppressing Th1 responses (80) and inducing FOXP3^+^ Tregs (120–122).

Figure 1 summarizes data from existing reports on the proposed mechanisms via which monocytes/macrophages may affect the function of CD4^+^ effector vs. Tregs subsets. It should be noted that much of the reported evidence comes from *in vitro* studies, which indicate the potential involvement of these mechanisms but do not demonstrate conclusively if or where these events occur at the site of inflammation.

## Effects of CD4^+^ T Cell Subsets on Monocyte/Macrophage Activation and Function

### Monocyte/Macrophage Activation and Polarization by CD4^+^ Effector T Cells

Monocytes and macrophages are capable of responding to a wide range of stimuli and environmental cues, which in turn can determine their phenotype. Macrophages can be polarized into diverse subtypes often termed M1 and M2; however, these phenotypes represent extremes on a spectrum of functional states (125). A recent transcriptomics study provided elegant evidence to support the latter concept (126). In this study, gene expression profiling was performed on 299 macrophage samples stimulated with 28 different conditions. Monocytes were differentiated into macrophages using either GM-CSF or M-CSF. The macrophages were subsequently activated using typical M1 (e.g., IFNγ, LPS, and TNF) or M2 (e.g., IL-4 and IL-13) stimuli, and standardized microarrays were performed. Analysis of the transcriptomes using coregulation analysis generated two groups of samples positioned at either end of a bimodal axis, representing M1/M2 states. However, when the effect of other activation factors not associated with typical M1/M2 states was included in the analysis (e.g., high-density lipoprotein and free fatty acids), the spectrum expanded away from the axis to account for these dissimilar states. These data confirm that while it is possible to polarize macrophages into distinct M1/M2 populations, not all activators will generate populations that fit into or in between these states. It is therefore important to keep in mind that although in this review we refer to M1/M2 macrophages, a spectrum of activation outside of these phenotypes exists. Outside of stringent M1/M2 phenotypes, it has become increasingly difficult to determine the exact nature of macrophages within published work. A recent review has highlighted a lack of coherently used markers used to define macrophage populations within the field (127). The authors discuss that incomplete descriptions of how macrophages have been isolated, stimulated, and analyzed can lead to confusion between laboratories. Therefore, they propose a new nomenclature system, based on the macrophage activator and markers used to define the population. In this way, the anticipation is that we can better define and understand the distinct macrophage populations generated in different labs under different conditions and avoid confusion when attempting to place macrophages on a spectrum of activation.

Bearing in mind the limitations of the M1/M2 classification, cytokines that are typically associated with Th1 and Th2 cells have been used to polarize monocytes or macrophages *in vitro* with the Th1 cytokine IFNγ driving the M1 (classically activated) phenotype and the Th2 cytokines IL-4/IL-13 driving an M2 (alternatively activated) phenotype (128). Three further M2 subtypes have since been described; M2a, M2b, and M2c, all with different functional capabilities to those seen in an M1 phenotype (127, 129). M1 macrophages typically release high levels of proinflammatory cytokines, such as IL-1, IL-6, IL-12, and TNFα, have high production of reactive oxygen intermediates and metabolize arginine to nitric oxide. Typical M1 chemokines include CXCL5, CXCL8, CXCL9, CXCL10, and CXCL13. M2 macrophages are characterized by a switch in arginine metabolism from the iNOS pathway seen in M1 cells to the arginase pathway, releasing orthonine and polyamines. M2 cells release IL-10 and some IL-12 and express CD163, CD206, scavenger receptors A and B, and Dectin-1. M2 macrophages are generally considered to be involved in wound healing and to promote tissue remodeling through release of growth factors VEGF and TGFβ (129, 130).

In addition to Th1 and Th2 cytokines, it has been shown that the Th17-associated cytokine IL-17A has direct effects on macrophages leading to an increase in IL-1β, TNFα, and IL-6 (131, 132). Silencing of IL-17RA using siRNA reduced the upregulation of IL-1β, TNFα, and IL-6 by macrophages, thus showing that IL-17 signaling through IL-17RA can influence cytokine release of macrophages (132). IL-17 has been shown to be directly chemotactic for monocytes *in vitro* at concentrations found in RA synovial fluid, via ligation of IL-17RA and IL-17RC on monocytes and p38 MAPK activation (133). *In vivo*, human monocytes injected intravenously into SCID mice were recruited into subcutaneously implanted sponges which had been soaked with human IL-17 or the positive control CCL2 [also called monocyte chemoattractant protein-1 (MCP-1)] but not sponges soaked with IL-8 or IL-10 (133). Tissue-infiltrating Th17 cells (unlike Th1 and Th2 cells) also secrete CCL20 (45), which has been shown to be chemotactic for monocytes (134). IL-17 may also have indirect effects on monocyte chemotaxis through the induction of chemokine expression by other cell types present in the RA joint. *In vitro* addition of IL-17 to RA synovial fibroblasts or normal blood-derived macrophages effectively induced expression of CCL2 and CCL20 (135). However, *in vivo* only CCL2 was secreted following adenovirus-mediated intraperitoneal (i.p.) expression of IL-17. The increase in peritoneal CCL2 levels contributed to increased monocyte recruitment, which was reduced by i.p. injection of neutralizing anti-CCL2 (135). Local IL-17 expression in ankle joints was also associated with increased F4/80 staining and CCL2 levels. The IL-17-mediated induction of CCL2 appeared to involve the PI3K, ERK, and (at least in RA synovial fibroblasts) JNK pathways.

There is good evidence that TCR- or cytokine-activated T cells can activate monocytes, resulting in inflammatory cytokine and MMP production by monocytes in a cell-contact-dependent manner (39, 136–138). The CD4^+^CD45RO^+^CCR7^−^ effector memory T cell subset of cytokine-activated T cells has been suggested to be a main driver of this stimulation (139). One study showed that distinct CD4^+^ T cell subsets (Th1, Th2, or Th17) may differentially affect monocyte differentiation into distinct mo-DC subsets in a cell-contact- and cytokine-dependent manner (42). Cocultures of sorted Th1, Th2, and Th17 cells with isolated CD14^+^ monocytes led to the generation of three distinct mo-DC subsets, as defined by typical DC markers. The monocytes cultured with Th1 cells formed DCs that secreted IL-12 and expressed CD86 and CD274 (DCth1), whereas those generated through culture with Th2 cells expressed increased levels of IL-10, CD275, and DC-SIGN (DCth2). Monocytes cultured with Th17 cells developed into DCs that secreted IL-1β, IL-6, and IL-23 but not IL-12 (DCth17). The DCs generated through coculture were then used as stimulators in a mixed leukocyte reaction; IFNγ and IL-17A were released from responding CD4^+^ T cells when cultured with DCth1 and DCth17, respectively. These data are in line with the results described above on how different Th-related cytokines affect macrophage polarization.

CD4^+^ T cells can also drive differentiation of monocytes into osteoclasts. Bone resorption by osteoclasts is of pathological significance in RA, causing “erosion sites” which can be used as a measure of disease severity and outcome. Studies have shown that IFNγ^+^ human T cells cultured with peripheral blood monocytes in the presence of M-CSF can induce osteoclast formation via expression of the cytokine receptor activator of NF-κB ligand (RANKL) (140). IFNγ may, however, also disrupt the formation of osteoclasts by rapidly degrading the RANK adaptor protein TRAF6 (141), suggesting that the IFNγ^+^ T cells can both contribute to and hinder the formation of osteoclasts. Th17 cells are often implicated in promoting osteoclastogenesis; Th17-associated cytokines were shown to upregulate RANKL on RA FLS and to directly induce monocyte-to-osteoclast differentiation (142, 143). In addition, RANKL-expressing Th17 cells were recently shown to convert mature osteoclasts to a bone resorptive state (144). T cells in the synovial fluid have been shown to express RANKL (145), and high levels of RANKL-expressing CD3^+^ cells have been found in the synovial tissue of patients with RA (146), thus potentially contributing to osteoclast formation and therefore higher levels of bone resorption.

Together, these data indicate that prototypical T helper cell-associated cytokines can polarize, recruit, activate, or differentiate monocytes and/or macrophages. *In vivo*, the phenotype and function of monocytes and macrophages is likely to be dependent on many soluble factors and cellular interactions acting in concert.

### Induction of Apoptosis in Monocytes and Macrophages by CD4^+^ T Cells

In addition to activating monocytes or macrophages, several reports have shown that activated CD4^+^ T cells can kill these cells (147, 148). Later studies assigned this killing to CD4^+^CD25^+^ Tregs (149, 150) as well as to activated effector T cells, defined as Treg-depleted CD4^+^CD25^−^ T cells or cloned antigen-specific CD4^+^ T cells (39, 148). A recent study showed that CD4^+^ (and CD8^+^) T cells from the BAL fluid of C57BL/6 mice express FASL and that these T cell populations can induce apoptosis in autologous alveolar macrophages. Although apoptosis by CD8^+^ T cells was more prevalent, killing by CD4^+^ T cells was observed (151). Evidence from our lab has shown that activated CD4^+^CD25^−^ effector T (Teff) cells upregulate FASL, and upon coculture with human monocytes, activate, and then kill the monocytes in a FAS/FASL-dependent manner (39). Blocking the FAS/FASL interactions reduced monocyte apoptosis but did not affect the expression of FAS, CD14, or HLA-DR on the monocytes, indicating that the monocytes still became activated by the T cells. T cells may also kill monocytes/macrophages in a FAS-independent mechanism, as TRAIL and TWEAK death receptor pathways have been implicated in macrophage killing by CD4^+^ T cells (152).

### Modulation of Monocyte/Macrophage Function by CD4^+^ Regulatory T Cells

Regulatory T cells are generally defined as CD4^+^CD25^+^CD127^low^FOXP3^+^ cells. The suppressive effects of Tregs on immune cells have been documented widely, in particular on cells from the adaptive immune system (CD4^+^ and CD8^+^ T cells) [reviewed in Ref. (153–155)]. Tregs can employ several mechanisms of suppression including release of the inhibitory cytokines IL-10 and TGFβ, cytolysis via release of granzymes A and B, metabolic disruption via IL-2 consumption or through degradation of ATP to AMP/ADP, and eventually adenosine via the ectonucleotidases CD39 and CD73. There is ample evidence that Tregs can also interact directly with antigen-presenting cells, including monocytes and macrophages [reviewed in Ref. (156)].

Modulation of monocyte function *in vitro* has been shown by a series of experiments from our lab. The first study compared monocytes from the peripheral blood of healthy human donors cultured alone, or cocultured with autologous effector T cells or Tregs. Coculture with anti-CD3 mAb and effector T cells induced an activated phenotype in the monocytes, with increased levels of CD80, CD40, and HLA II compared to monocytes cultured alone (157). When monocytes were cocultured with Tregs, levels of CD40, CD80, and HLA II on monocytes were not increased compared to the monocyte only culture, while CD86 expression was significantly reduced. These monocytes were impaired in their ability to induce T cell proliferation in subsequent T cell stimulation assays. In a following study, we demonstrated that upon coculture with Tregs, monocytes expressed increased levels of the mannose receptor CD206 and hemoglobin/haptoglobin scavenger receptor CD163 (158), markers which are associated with M2-like macrophages (127, 129). In support of these phenotypic changes, we found that phagocytosis of FITC-zymosan/latex beads was increased in Treg cocultured monocytes, although the number of monocytes that phagocytosed the beads did not change significantly (158). Furthermore, LPS-induced NFκB activation and secretion of TNFα and IL-6 were decreased in monocytes cocultured with Tregs, compared to those cultured alone or with effector T cells. Using flow cytometry, it was shown that the frequencies of LPS-induced IL-6 and TNFα expressing monocytes were also reduced upon coculture with Tregs as compared to monocytes cultured alone or with Teffs, indicating that the observed differences in cytokine secretion were due to changes within cytokine-expressing monocytes (39). This was not due to cell death, as in contrast to CD4^+^CD25^−^ Teff, Tregs did not kill the monocytes upon interaction (39). The decreased production of IL-6 and TNFα in response to LPS was still observed when monocytes were re-purified after coculture (158), indicating that Tregs imprint changes in the monocytes, suggestive of “trained immunity.” Immune memory has traditionally been associated with cells of the adaptive immune system; recently, the term “trained immunity” has been used to define the memory capacity within innate immune cells (159). Induction of trained immunity in monocytes has been shown in studies whereby exposure to beta-glucan led to epigenetic modifications in monocytes, resulting in phenotypic changes and altered function (160). Further investigation is required to determine whether the effects of Tregs on monocytes are reflective of a state of “trained immunity” in these cells.

The modulation of monocytes by Tregs was shown to be dependent in part on soluble factors (IL-10 and IL-4/IL-13) as well as cell-contact (158). A different group showed that cocultures of human CD14^+^ monocytes with sorted Tregs led to increased levels of secreted IL-10 and higher levels of B7-H4 receptor on the monocytes. These monocytes were less capable of stimulating a T cell proliferative response, which was due in part to the expression of IL-10 and B7-H4 (161). Notably, work from the same group showed that the expression of B7-H4 was a marker for immunosuppressive tumor-associated macrophages in ovarian carcinoma (162), and that the presence of Tregs and macrophage-associated B7-H4 at the tumor site was negatively associated with patient outcome (163, 164). Together, these data indicate that modulation of monocytes and macrophages by Tregs may have functional consequences in disease.

Modulation of macrophage function by Tregs has also been shown *in vivo*. Adoptive transfer of syngeneic CD4^+^CD25^+^ Tregs into the peritoneal cavity of SCID mice revealed both phenotypic and functional changes in peritoneal macrophages (165). In this study, SCID mice were adoptively transferred with either no T cells, CD4^+^CD25^+^ Tregs, CD4^+^CD25^−^ T cells, or both CD4^+^CD25^+^ Treg and CD4^+^CD25^−^ T cells at a 1:1 ratio. The percentages of F4/80^+^ macrophages expressing CD54, CD80, CD86, or I-A^d^ were significantly decreased in SCID mice transferred with Tregs compared to the control mice and were increased in mice receiving CD4^+^CD25^−^ T cells. The latter increase was inhibited when both T cell types were cotransferred. The group also showed that macrophages from mice transferred with Tregs had an enhanced phagocytic capacity compared to those transferred with CD4^+^CD25^−^ T cells or no T cells. The enhanced phagocytic capacity was reversed by transfer with both cell types. Upon LPS stimulation, macrophages from SCID mice transferred with CD4^+^CD25^+^ Treg cells produced more IL-10 and less IL-12 than those from mice transferred with CD4^+^CD25^−^ T cells. The macrophages from mice receiving Tregs were also impaired in their antigen-presenting capacity and showed higher arginase activity and lower nitric oxide production compared to those from mice transferred with CD4^+^CD25^−^ T cells or no T cells. Together, this work suggests that Tregs modulate macrophages toward an “M2” phenotype, with the functional and phenotypic characteristics of the modulated cells closely resembling those seen in the previously mentioned *in vitro* studies of human Tregs and monocytes.

**Figure 2:**
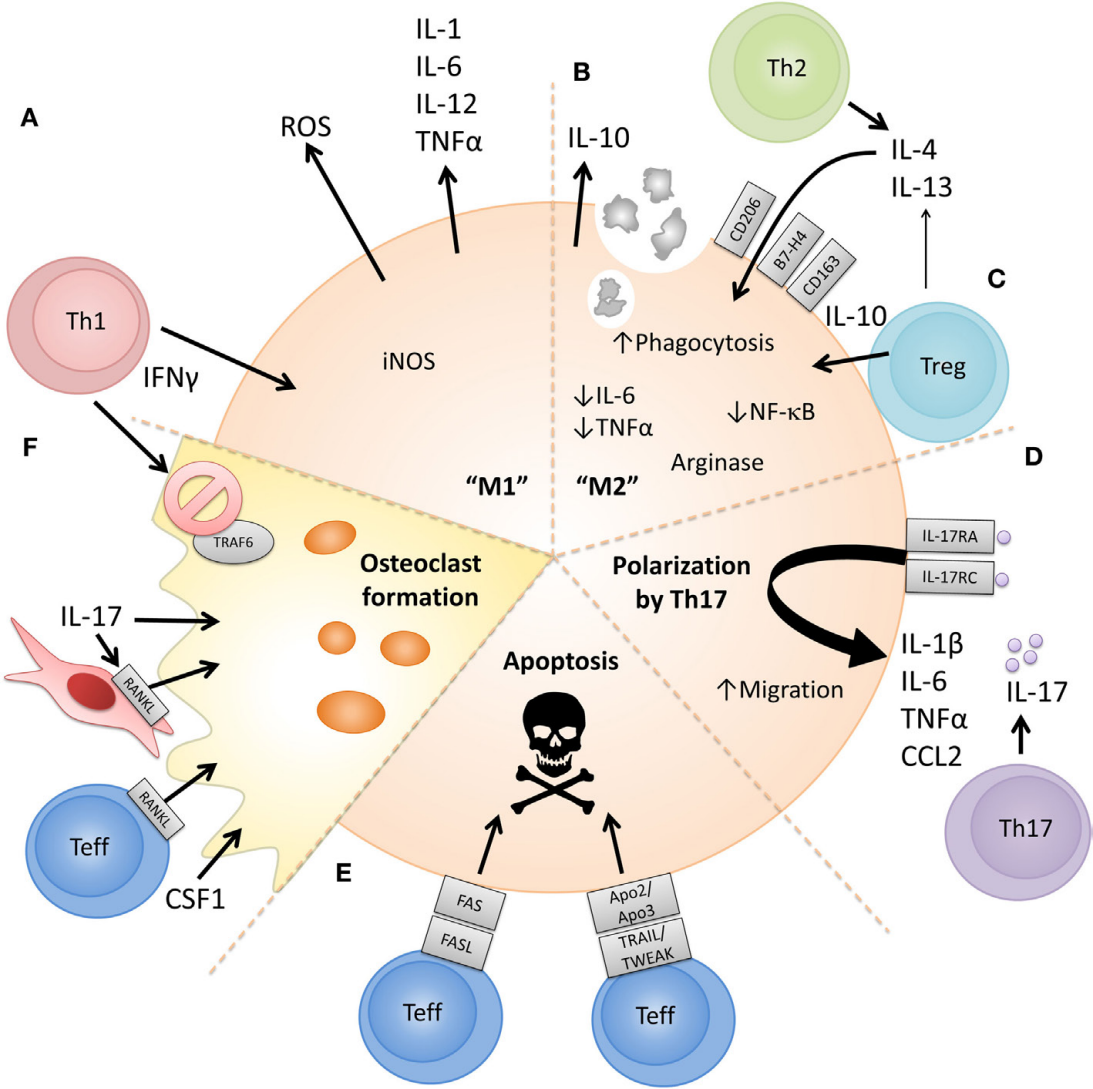
**Proposed cellular and molecular mechanisms via which CD4^+^ T cell subsets can polarize or modulate monocyte/macrophage function**. **(A)** The prototypical Th1 cytokine IFNγ can polarize monocytes to an “M1” phenotype, promoting proinflammatory cytokine release, reactive oxygen species (ROS) production, and metabolism of arginine to nitric oxide [reviewed in Ref. (125, 127)]. **(B)** Prototypical Th2 cytokines IL-4 and IL-13 can polarize monocytes to an “M2” phenotype, characterized by expression of anti-inflammatory cytokines such as IL-10, expression of CD206, increased phagocytosis, and metabolism of arginine to orthonine and polyamine intermediates [reviewed in Ref. (125, 127)]. **(C)** CD4^+^CD25^+^ Tregs have also been shown to induce an alternatively activated or anti-inflammatory phenotype in monocytes in a cell-contact and soluble factor (IL-4, IL-13, and IL-10) dependent manner leading to reduced IL-6 and TNFα production, decreased NFκB activation and increased expression of IL-10, CD163, and B7-H4 (157, 158, 164, 165). **(D)** The prototypical Th17-associated cytokine IL-17 has been shown to increase levels of IL-1β, TNFα, and IL-6 released by macrophages (131, 132). IL-17 has also been suggested to be chemotactic for monocytes via ligation of IL-17RA/RC (133). **(E)** Evidence shows that activated CD4^+^CD25^−^ T cells can activate but also induce apoptosis in monocytes/macrophages. Both FAS/FASL- and TRAIL/TWEAK-dependent mechanisms have been proposed (39, 147, 148, 151, 152). **(F)** Activated RANKL-expressing Teff can drive osteoclast formation from monocytes when cultured in the presence of M-CSF (140). Th17-associated cytokines can upregulate RANKL on RA fibroblast-like synoviocytes and also directly induce monocyte-to-osteoclast differentiation (142, 143), while IFNγ has been shown to suppress osteoclast formation (141).

Figure 2 summarizes existing evidence on the cellular and molecular mechanisms via which CD4^+^ effector vs. regulatory T cell subsets can affect the phenotype or function of monocytes and macrophages. As indicated in Figure 1, since much of the reported evidence comes from *in vitro* studies, some caution should be exerted when extrapolating these findings to an *in vivo* situation.

## Dysregulated Monocyte/Macrophage Homeostasis in the Rheumatoid Joint

The abundance of CD14^+^/CD68^+^ monocytes/macrophages in the rheumatoid joint suggests that these cells are recruited at a high rate, are long-lived or proliferative, and/or resistant to apoptosis. Evidence exists to support all these scenarios. Chemokines involved in monocyte recruitment, such as CCL2/MCP-1, CCL3/MIP-1α, and CCL5/RANTES, are readily detectable at the site of inflammation (96, 166), and blood monocytes from patients with RA express the corresponding chemokine receptors CCR1, CCR2, CCR3, and CCR5 (15). One study investigated the migration of labeled autologous CD14^+^ blood monocytes, isolated by CliniMACS procedure, in RA patients using single photon emission computer tomography. A very small but specific fraction of 0.003% of re-infused monocytes was found to migrate to the inflamed joints, being detectable within 1 h after re-infusion (167). Interestingly, monocyte influx into the inflamed joint was not altered early after anti-TNF treatment (2 weeks post-treatment) even though disease activity was significantly reduced (168). The authors concluded that monocytes migrate continuously into the inflamed synovial tissue of RA, but at a slow macrophage-replacement rate, and that the rapid decrease in synovial macrophage numbers observed after anti-TNF treatment (23) cannot be explained by an immediate effect on monocyte influx.

Another contributing factor to the persistently high monocyte/macrophage presence in the rheumatoid joint is apoptosis resistance. Both RA peripheral blood monocytes and RA synovial monocytes/macrophages have been shown to be resistant to spontaneous cell death, agonistic Fas-antibody induced apoptosis, or responder T cell-mediated killing (28, 39, 40). Proposed underlying mechanisms include the enhanced expression of antiapoptotic molecules, such as FLIP and Mcl-1 in RA synovial tissue (169, 170), the reduced expression of the proapoptotic Bcl-2 homology 3 (BH3)-only protein Bim (171), the increased production of TNFα and IL-1 which have antiapoptotic effects (172), and the increased presence of Tregs in the RA joint (173), which do not exert apoptotic effects on monocytes (39). Incubation with the anti-TNF drugs infliximab or adalimumab has been shown to increase apoptosis in healthy or RA peripheral blood monocytes, lending support to the role of TNF in regulating this process (28, 174). Conversely, defects in apoptosis pathway-associated molecules, such as occur in Fas-deficient (*lpr/lpr*) mice, lead to an increase in the number of circulating monocytes and an increase in the proinflammatory activity of peritoneal macrophages and the development of systemic autoimmune disease including lupus-like disease and inflammatory arthritis (175). A recent study showed that selective loss of Fas in myeloid cells was sufficient to induce SLE-like disease in mice (176). Together, these data illustrate that a dysregulation in monocyte/macrophage homeostasis may be an important contributing factor to chronic inflammatory joint disease.

In addition, recent advances in genetics have revealed an increasing number of susceptibility loci for RA (177), several of which may have relevance to monocyte/macrophage function or homeostasis, e.g., *CD40*, *TNFAIP3*, *IRAK1*, *TRAF1/TRAF6*, *IRF5* (178), and *RBPJ* (179). Future functional genomic studies may elucidate the exact role of these genes in RA and in monocytes/macrophages in particular.

Although knowledge regarding the phenotype and function of synovial macrophages is continuing to grow, it is much less clear what the origin of these cells is. Fate-mapping experiments in mice demonstrate that tissue-resident yolk sac-derived macrophages develop in a Myb-independent manner and can persist in adult mice independently of hematopoietic stem cells (180, 181). However, other work suggests that Myb-dependent fetal liver-derived monocytes also contribute to the pool of tissue-resident cells (182, 183). In addition, although self-renewing tissue-resident macrophage populations have been described (182–185), evidence suggests that recruited blood monocytes may play a role in replenishing the macrophage population during inflammation (186–188). The investigation of the origin and homeostasis of tissue macrophages remains an active field of research, and future work may be able to determine whether RA synovial macrophages are equivalent to these long-lived cells, or are continuously replaced by newly recruited peripheral monocytes.

## Targeting Monocytes/Macrophages in RA

The findings discussed in this review indicate that selective targeting of monocytes/macrophages could have therapeutic benefit in RA [also reviewed in Ref. (5)]. This is supported by older data showing that treatment of severe RA with leukapheresis efficiently removed blood monocytes concomitant with a clinical response (189), and that depleting CCR2^+^ monocytes using anti-CCR2 mAb could ameliorate collagen-induced arthritis, although the effects were dependent on the dose of mAb used (190). *In vitro*, selective elimination of human macrophages using toxin-conjugated Abs against CD64 resulted in reduced T cell proliferation and reduced TNFα production by synovial fluid mononuclear cells and synovial tissue explants (191). Bim-BH3 mimetic therapy could induce apoptosis in myeloid cells and suppress clinical severity of experimental arthritis (171). siRNA-based therapeutic approaches are also being developed in order to selectively target certain genes or pathways (192).

Additionally, given the importance of T cell-monocyte crosstalk in promoting inflammation (17, 137, 193), approaches that target T cell-monocyte interactions may have therapeutic benefit. Indeed, this may be one way via which drugs like abatacept (CTLA4-Ig) exert their clinical effect. Previously underappreciated mechanisms of existing therapeutics may contribute to their efficacy by (in)directly targeting monocytes/macrophages. For example, multiple studies have shown that blockade of IL-6 signaling via monoclonal antibody to the IL-6 receptor (tocilizumab) can boost Treg frequencies (194–198), but additional mechanisms of action may include reduction in CD69^+^CD14^+^ and CD16^+^CD14^+^ monocyte frequencies (197), induction of monocyte apoptosis, and inhibition of monocytic IL-6 mRNA expression (199). Despite the widespread uptake and high efficacy of TNFα inhibitor drugs in RA, the underlying mechanisms of action are not firmly established [reviewed in Ref. (200)]. TNF inhibitor drugs block signaling of the key monocyte/macrophage-derived cytokine TNFα but can also reduce production of other proinflammatory mediators, such as IL-1β, GM-CSF, IL-6 and IL-8, in synovial membrane (201–203). Furthermore, it has been reported that Treg function can be restored or enhanced following TNFα inhibitor therapy (111, 204), and we and others recently demonstrated that TNFα blockade in cocultures of antigen presenting cells and CD4^+^ T cells favors development of an immunoregulatory phenotype in effector CD4^+^ T cells by promoting expression of IL-10 (205, 206). We showed that following TNF blockade, a significant proportion of IL-17 expressing CD4^+^ T cells coexpressed IL-10, which was biologically active and was able to modulate the phenotype of monocytes leading to reduced HLA-DR and CD40 expression and increased CD163 expression (205). Another postulated mechanism of anti-TNF drugs is via interaction with tmTNFα (28, 207). It was recently shown that production of cytokines and decoy receptors triggered by monocyte tmTNF crosslinking might provide a prognostic parameter for predicting therapeutic response to etanercept, suggesting a role for monocytic reverse signaling in the clinical efficacy of TNF inhibitors (208).

As previously discussed, monocytes/macrophages contribute to bone erosion in RA via differentiation into osteoclasts. Inhibiting osteoclastogenesis therefore presents a key target for therapeutic intervention, since the presence of bone erosions represents irreversible structural damage, associated with loss of joint function and poor quality of life. TNFα inhibitor drugs inhibit radiological progression, even in the absence of clinical response, via direct inhibitory effects on osteoclast differentiation and activity (209). Binding of CTLA4 to CD80/86 on monocytes provides a potent signal to inhibit differentiation into osteoclasts; accordingly abatacept was recently shown to inhibit osteoclastogenesis in human peripheral monocytes (210). A phase II clinical trial found that twice-yearly injections of the anti-RANKL drug denosumab inhibited progression of bone erosion in RA patients with active erosive disease but demonstrated no effect on disease activity (211). Similarly, RANKL knockout mice are protected from bone erosion in a serum transfer model of arthritis but display inflammation that is clinically and histologically similar to wild type (212). These data suggest that targeting osteoclastogenesis may have the potential to reduce structural joint damage, but that RANKL blockade alone may be ineffective at controlling the underlying inflammatory process. As an alternative strategy to inhibit osteoclastogenesis, targeting M-CSF receptor (M-CSFR, also known as CSF1R, c-FMS, and CD115) has been shown in various animal models of autoimmune arthritis to reduce both joint inflammation and bone destruction (213–216) However, in some models, while bone erosion is reduced, inflammation is unaffected by M-CSFR antibody blockade (215) or c-FMS kinase inhibition (217). Effective targeting of the M-CSFR pathway in RA may require blocking signaling of both its ligands, M-CSF and IL-34. Several clinical trials are currently investigating antibodies or small molecules targeting the M-CSFR pathway in RA and other indications [reviewed in Ref. (218)].

Epigenetic control of immune-mediated processes is a growing field of study with the potential to unveil mechanisms of immune regulation which may be amenable to therapeutic intervention. Bromodomain and extraterminal (BET) inhibitors have recently emerged as a promising approach to treat cancer but are also being investigated in the context of inflammatory disease. Blocking the recruitment of BET proteins to acetylated histones inhibits BET-mediated transcriptional activity. BET inhibitors have been shown to suppress expression of proinflammatory cytokines and chemokines in LPS-stimulated bone marrow-derived macrophages *in vitro* (219, 220). *In vivo* BET inhibition resulted in reduced Th17 differentiation, suppression of established Th17 responses, and protection against pathology in collagen-induced arthritis (221). A recent study found that BET inhibition suppressed cytokine-induced transcription in primary human monocytes in a gene-specific manner, without affecting JAK-STAT signaling. Instead, BET inhibition reduced recruitment of transcriptional machinery to the CXCL10 promoter and an upstream enhancer (222). In future studies, global approaches, such as genome-wide profiling, may identify additional functional pathways that are amenable to BET-mediated transcriptional regulation in monocytes/macrophages.

Additional therapeutic approaches under development for RA include proteasome inhibitors, such as bortezomib (223). Conflicting data on the effects of bortezomib on bone resorption are reported in different animal models of RA (224, 225). In human cells, bortezomib appears to inhibit osteoclastogenesis (226, 227). Selective inhibition of a subunit of the immunoproteasome (a class of proteasome primarily found in monocytes and lymphocytes) was shown to inhibit production of IL-23, TNFα, and IL-6 by LPS-activated monocytes and to reduce IFNγ and IL-2 production from anti-CD3/CD28-activated T cells (228). These data were generated using cells from healthy donors, but similar results were observed using PBMC from three RA patients. In mouse models of RA, immunoproteasome inhibition ameliorated disease and also blocked IL-23 production from activated monocytes.

Protein kinase inhibitors are another class of small molecule therapeutics gaining attention for the treatment of immune-mediated diseases. Tofacitinib, the first Janus kinase (JAK) inhibitor to be developed for RA was approved by the FDA in 2012. To date, most studies have focused on the effects of JAK inhibition in T cells with relatively few data on the consequences in monocytes/macrophages. A recent report found that tofacitinib did not directly affect RA synovial monocytes, but that tofacitinib-exposed CD4^+^ T cells demonstrated reduced proliferation, impaired IL-17 and IFNγ expression, and generated conditioned medium that when added to CD14^+^ monocytes could inhibit IL-8 production (229). Others showed that tofacitinib reduced CD80/CD86 expression and therefore the T cell costimulatory capacity of human mo-DCs (230). In addition, tofacitinib and ruxolitinib, another JAK inhibitor drug, were shown to effectively suppress inflammatory responses of blood-derived and RA synovial macrophages (231). Tofacitinib also efficiently suppressed development of arthritis in a K/BxN serum transfer model. A cautionary observation was that JAK inhibition could enhance osteoclast differentiation *in vitro*. Also, in isolated mouse macrophages, both tofacitinib and ruxolitinib were found to block the IL-10-mediated feedback inhibition of cytokine transcription, thereby increasing LPS-induced cytokine production (232). These data indicate that JAK inhibitor drugs do have the capacity to modulate monocyte/macrophage function, but the exact cellular mechanisms that mediate the clinical efficacy of these therapeutics are still under investigation.

**Figure 3:**
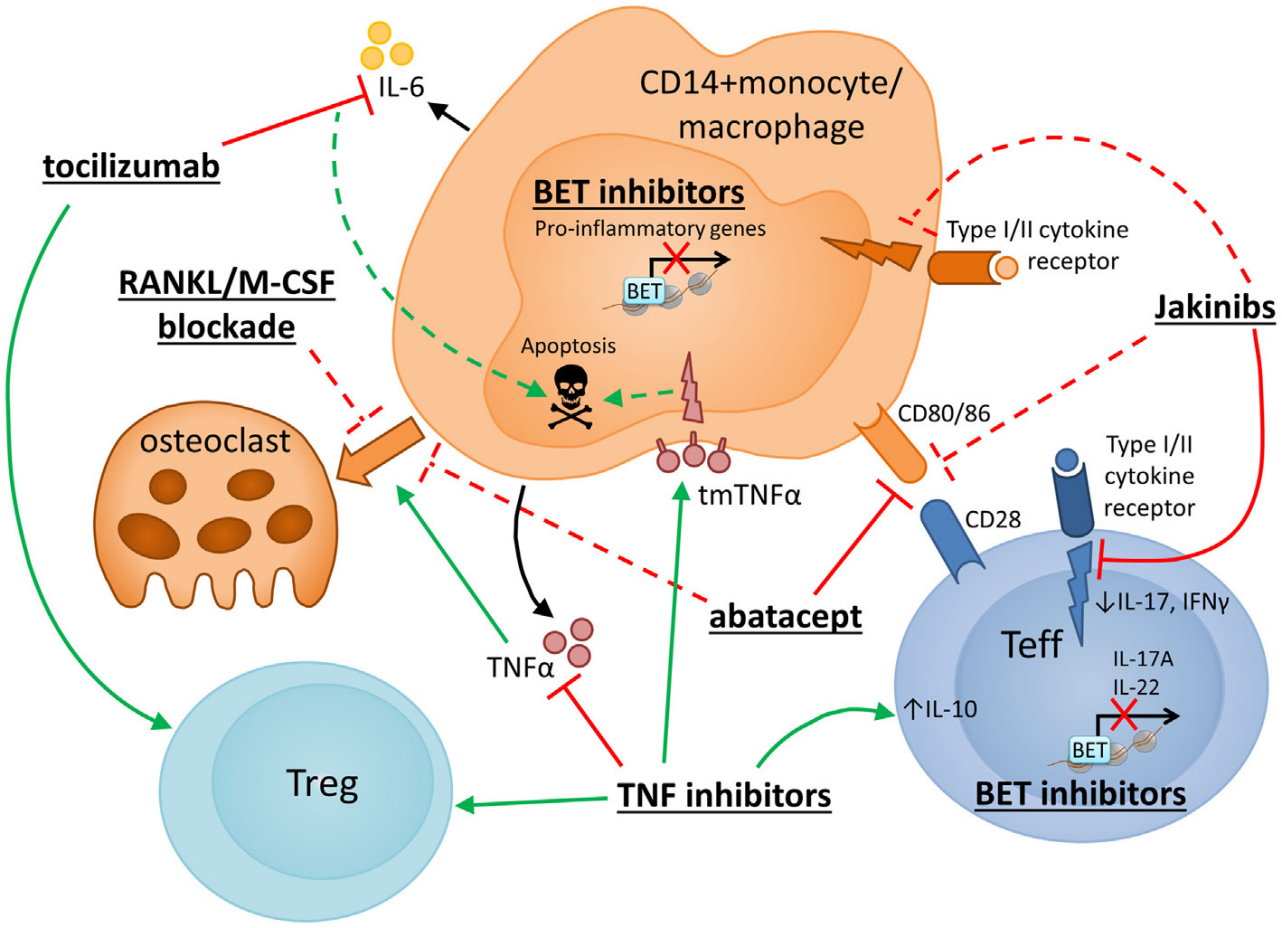
**Therapeutic strategies to target monocytes/macrophages in RA**. Several novel strategies for drug intervention in RA, as well as currently used therapeutics, have potential mechanisms of action that target monocyte/macrophages or their interaction with CD4^+^ T cells. Selected therapies are presented with particular reference to their reported effects on monocyte/macrophage function and CD4^+^ T cell interactions. Red and green lines indicate inhibition and promotion, respectively, while dashed lines indicate novel or putative mechanisms that may require further confirmation. TNF inhibitor drugs block signaling of the key monocyte/macrophage-derived cytokine TNFα and have been shown to promote increased IL-10 expression in CD4^+^ T cells (205, 206), enhanced or restored Treg function (111, 204) and anti-osteoclast effects (209), and may also exert anti-inflammatory/proapoptotic effects on monocytes via “reverse signaling” through tmTNFα (28, 208). Blockade of IL-6 signaling (via monoclonal antibody to the IL-6 receptor, tocilizumab) reportedly boosts Treg frequencies (194–198) and has been suggested to inhibit monocyte IL-6 mRNA expression and induce monocyte apoptosis (199). Another widely used RA drug, abatacept (CTLA4-Ig), targets the interaction between monocytes and T cells, specifically impairing T cell costimulatory signals via CD80/CD86. Abatacept has also been reported to inhibit monocyte differentiation into osteoclasts (210). Several other approaches are currently under development to specifically target osteoclastogenesis, including blockade of RANKL (211) or M-CSF (218). Janus Kinase (JAK) inhibitors (Jakinibs) target JAK/STAT-mediated cytokine signaling in T cells and possibly also in macrophages (231, 232) and may reduce monocyte-derived DC costimulatory capacity (230). Bromodomain and extraterminal (BET) inhibitors are also under consideration for the treatment of inflammatory disease. Efficacy has been shown in collagen-induced arthritis where BET inhibition reduced Th17 responses (221). Reduced transcription of proinflammatory genes has been described in human monocytes (222) and mouse macrophages (219, 220) following *in vitro* exposure to BET inhibitors.

Figure 3 shows an overview of how drugs currently used to treat RA, as well as therapeutics under development, may target monocytes/macrophages or their interaction with CD4^+^ T cells, to intervene in the underlying inflammatory and erosive disease processes.

## Summarizing Conclusion

In summary, there is strong evidence for a contributing role of both monocytes/macrophages and CD4^+^ T cells in RA. In addition to directly promoting local inflammation by secreting proinflammatory mediators, synovial monocytes/macrophages secrete chemokines that can attract and maintain CD4^+^ T cells in the joint. A growing evidence base suggests that activated (subsets of) monocytes can influence CD4^+^ T helper cell polarization toward Th1/Th17. Through their cytokine production monocytes/macrophages may also impact on frequencies and function of regulatory CD4^+^ T cells. Conversely, CD4^+^ effector T cells can activate, polarize, as well as kill monocytes and macrophages and may influence monocyte chemotaxis, while CD4^+^ Tregs can exert immunomodulatory effects on these cells, thereby enhancing their survival and inducing an anti-inflammatory state in monocytes/macrophages. To inform future research, refining the phenotypic characterization of monocyte/macrophage subpopulations by validating additional phenotypic markers would facilitate further investigation of their involvement in inflammatory conditions, such as RA. Given that the complexity of monocyte/macrophage phenotypes is not well understood or necessarily reflected in animal models or immortalized cell lines, studies of human primary monocytes and macrophages are essential to understand the contribution of these cells to RA pathogenesis. Improved knowledge regarding the origin of synovial monocyte/macrophages could lead to a better understanding of the roles of tissue-resident macrophages in the RA joint. Finally, further investigation into the direct interactions between tissue-resident CD4^+^ T cell subsets and macrophages may elucidate how effector T cell responses are generated *in situ* as well as how macrophage responses can be regulated differentially by CD4^+^ effector and Tregs. Several existing RA therapeutics are known to impact on the crosstalk between monocytes and CD4^+^ T cells. An increased understanding of how the interactions between these cell types may contribute to immune pathology will benefit the development of new and improved therapeutic strategies.

## Conflict of Interest Statement

Leonie S. Taams has received research support from Novo Nordisk, UCB, and GSK. Ceri A. Roberts and Abigail K. Dickinson have no conflict of interest to declare.
